# The ability of TNPO3-depleted cells to inhibit HIV-1 infection requires CPSF6

**DOI:** 10.1186/1742-4690-10-46

**Published:** 2013-04-26

**Authors:** Thomas Fricke, Jose Carlos Valle-Casuso, Tommy E White, Alberto Brandariz-Nuñez, William J Bosche, Natalia Reszka, Robert Gorelick, Felipe Diaz-Griffero

**Affiliations:** 1 Department of Microbiology and Immunology, Albert Einstein College of Medicine Bronx, 1301 Morris Park – Price Center 501, New York, NY 10461, USA; 2 AIDS and Cancer Virus Program, SAIC-Frederick, Inc., Frederick National Laboratory for Cancer Research, Frederick, MD 21702, USA

## Abstract

**Background:**

Expression of the cellular karyopherin TNPO3/transportin-SR2/Tnp3 is necessary for HIV-1 infection. Depletion of TNPO3 expression in mammalian cells inhibits HIV-1 infection after reverse transcription but prior to integration.

**Results:**

This work explores the role of cleavage and polyadenylation specificity factor subunit 6 (CPSF6) in the ability of TNPO3-depleted cells to inhibit HIV-1 infection. Our findings showed that depletion of TNPO3 expression inhibits HIV-1 infection, while the simultaneous depletion of TNPO3 and CPSF6 expression rescues HIV-1 infection. Several experiments to understand the rescue of infectivity by CPSF6 were performed. Our experiments revealed that the HIV-1 capsid binding ability of the endogenously expressed CPSF6 from TNPO3-depleted cells does not change when compared to CPSF6 from wild type cells. In agreement with our previous results, depletion of TNPO3 did not change the nuclear localization of CPSF6. Studies on the formation of 2-LRT circles during HIV-1 infection revealed that TNPO3-depleted cells are impaired in the integration process or exhibit a defect in the formation of 2-LTR circles. To understand whether the cytosolic fraction of CPSF6 is responsible for the inhibition of HIV-1 in TNPO3-depleted cells, we tested the ability of a cytosolic full-length CPSF6 to block HIV-1 infection. These results demonstrated that overexpression of a cytosolic full-length CPSF6 blocks HIV-1 infection at the nuclear import step. Fate of the capsid assays revealed that cytosolic expression of CPSF6 enhances stability of the HIV-1 core during infection.

**Conclusions:**

These results suggested that inhibition of HIV-1 by TNPO3-depleted cells requires CPSF6.

## Background

TNPO3, transportin-SR2 or Tnp3 is a member of the karyopherin β superfamily of proteins, and works as a nuclear import receptor for serine-arginine-rich (SR) proteins, which are necessary for RNA splicing. It is an established fact that depletion of TNPO3 decreases the ability of wild type HIV-1 and other lentiviruses to infect cells [1-10]; however, the mechanism by which TNPO3 assists HIV-1 replication is under intense investigation with existing evidence to support a role for TNPO3 after reverse transcription but prior to integration [1-3,5,11].

Genetic and biochemical evidence suggests that the HIV-1 capsid is the viral determinant for the requirement of TNPO3 during infection [1,6,12,13]. One of the most important pieces of evidence supporting this notion is an HIV-1 virus bearing the capsid mutation N74D (HIV-1-N74D), which results in a virus insensitive to the depletion of TNPO3 [1,3,6,12]. Interestingly, the capsid mutation N74D was isolated by serial passage of HIV-1 viruses in human T-cells expressing a fragment derived from the cleavage and polyadenylation specificity factor subunit 6 (CPSF6) protein, which blocks HIV-1 infection before nuclear import [12]. The fact that HIV-1-N74D is insensitive to TNPO3-depletion and overcomes the restriction imposed by a fragment derived from CPSF6 suggests a role for CPSF6 in the ability of TNPO3-depleted cells to block HIV-1 infection.

The fragment derived from CPSF6 composed of residues 1-358 (CPSF6-358) localizes to the cytoplasm and potently restricts HIV-1 infection when overexpressed in different mammalian cells [12]. By contrast the full-length CPSF6 is a nuclear protein that when overexpressed in mammalian cells does not block lentiviral infection [12]. Because overexpression of CPSF6-358 blocks HIV-1 infection before nuclear import, CPSF6-358 might be interacting with the incoming viral core. In agreement with this notion, CPSF6-358 contains an HIV-1 capsid-binding domain [14].

This work tested the role of CPSF6 in the ability of TNPO3-depleted cells to inhibit HIV-1 infection. Depletion of TNPO3 expression inhibits HIV-1 infection; however, the simultaneous depletion of TNPO3 and CPSF6 expression rescues HIV-1 infectivity indicating that CPSF6 is required for the ability of TNPO3-depleted cells to block HIV-1 infection. To further understand the contribution of CPSF6, we tested the binding of endogenously expressed CPSF6 to the HIV-1 capsid extracted from wild type and TNPO3-depleted HeLa cells; these experiments revealed no difference in binding. CPSF6 localization studies in TNPO3-depleted cells showed that CPSF6 did not change localization. Because depletion of TNPO3 prevents HIV-1 infection after nuclear import, we studied the formation of 2-LTR circles blocking the enzymatic activity of HIV-1 integrase by genetic or pharmacological means. Our studies revealed that TNPO3-depleted cells are impaired in the integration process or exhibit a defect in the formation of 2-LTR circles. Because TNPO3-depleted cells inhibit HIV-1 infection in a CPSF6-dependent manner, we tested whether TNPO3-depleted cells inhibit HIV-1 infection by the mechanism used by CPSF6-358. For this purpose, we tested the ability of a cytosolic full-length CPSF6 to block HIV-1 infection. These results demonstrated that overexpression of a cytosolic full-length CPSF6 blocks HIV-1 infection at or before the nuclear import step. Overall these results suggested that inhibition of HIV-1 by TNPO3-depleted cells is CPSF6-dependent.

## Results

### Inhibition of HIV-1 infection by depletion of TNPO3 requires expression of CPSF6

**Figure 1:**
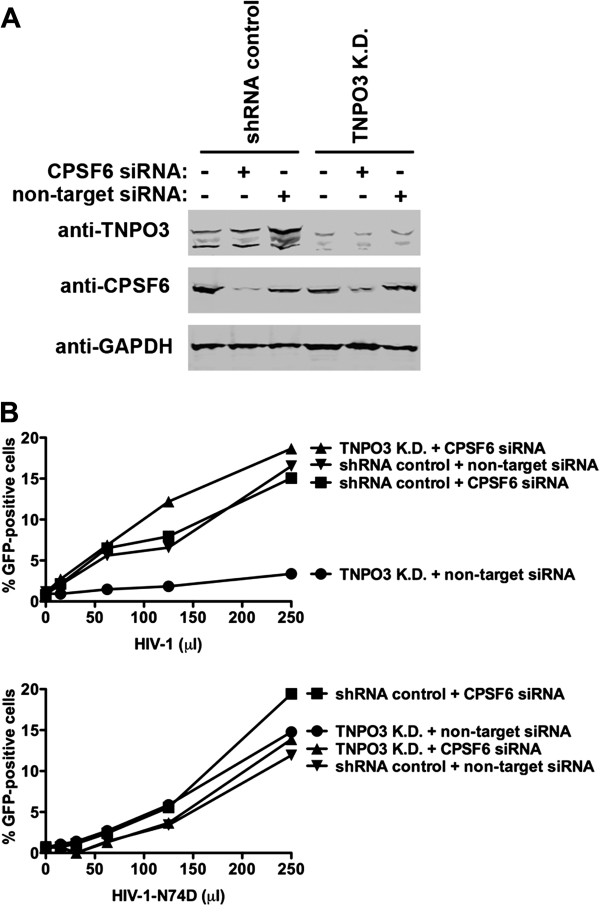
**Inhibition of HIV-1 infection by TNPO3-depleted cells requires expression of CPSF6.** HeLa TNPO3 K.D. and shRNA control human HeLa cells were transfected with a specific siRNA against CPSF6, a non-target siRNA, or left untreated. Forthy-eight hours after transfection, cells were lysed, and the expression levels of CPSF6 and TNPO3 were monitored by Western blot using antibodies against CPSF6and TNPO3, respectively (**A**). As a loading control, cell lysates were Western blotted against GAPDH. Fluorescence quantification revealed a 20 and 18 fold reduction in the expression of CPSF6 when using siRNA against CPSF6 in shRNA and TNPO3 K.D. cells, respectively. TNPO3 K.D. and shRNA control cells were challenged with increasing amounts of HIV-1 or HIV-1-N74D expressing GFP as a reporter for infection (**B**). Viruses were normalized by quantifying the particle-associated reverse transcriptase activity on viral supernatants, as described in Methods. Infectivity was determined forty-eight hours post-infection by measuring the percentage of GFP-positive cells using a flow cytometer. Similar results were obtained in three independent experiments and a representative experiment is shown.

To understand the role of CPSF6 in the ability of TNPO3-depleted HeLa cells to inhibit HIV-1 infection, we measured HIV-1 infectivity in HeLa cells simultaneously silenced for the expression of TNPO3 and CPSF6 (Figure 1). Initially, we stably knockdown the expression of TNPO3 by stably transducing HeLa cells with a specific shRNA against TNPO3 [1]. As shown in Figure 1A, transfection of TNPO3 K.D. cells by a specific siRNA against CPSF6 decreased the expression CPSF6 by 20-fold when compared to the non-target siRNA. Similarly, transfection of HeLa control cells stably transduced with the empty shRNA vector (pLKO.1) by siRNA against CPSF6 decreased expression by 20-fold when compared to non-target siRNA.

Next we tested the ability of HIV-1 to infect HeLa cells that are simultaneously silenced for the expression of TNPO3 and CPSF6. As shown in Figure 1B, depletion of TNPO3 and CPSF6 completely rescues HIV-1 infection when compared to TNPO3 K.D. HeLa cells transfected with a non-target siRNA. As expected TNPO3 K.D. HeLa cells potently inhibit HIV-1 infection [1]. Interestingly, depletion of CPSF6 alone did not affect HIV-1 infection (Figure 1B). As control, we challenged the different knockdown cells with HIV-1-N74D, a TNPO3-independent virus [1,3,6,12]. These control experiments revealed that simultaneous depletion of TNPO3 and CPSF6 does not affect HIV-1-N74D infection (Figure 1B). Overall, these experiments indicated that CPSF6 is required for the ability of TNPO3-depleted cells to inhibit HIV-1 infection.

### Binding of endogenously expressed CPSF6 to HIV-1 capsid in the absence of TNPO3

**Figure 2:**
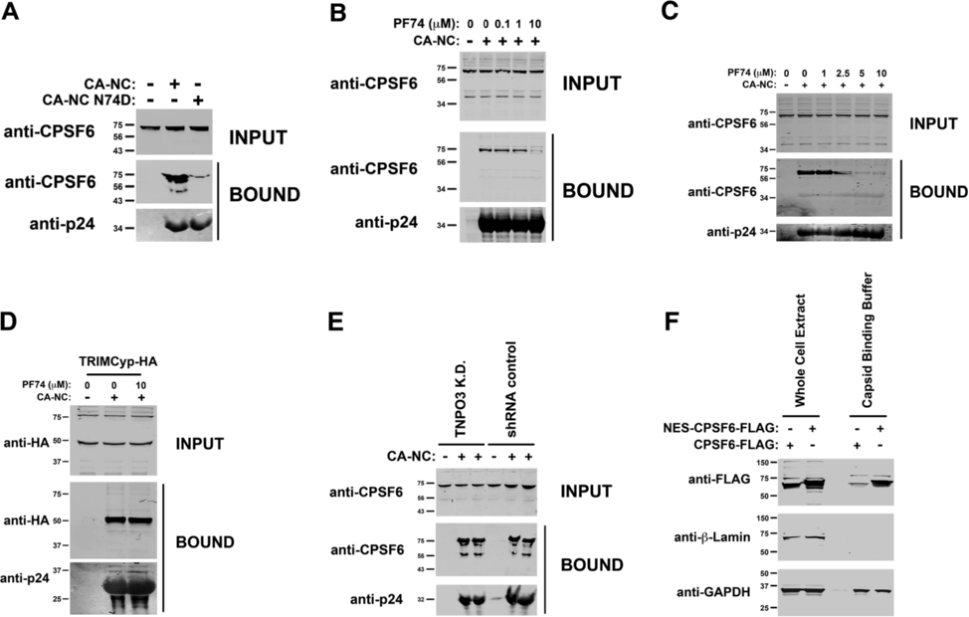
**Binding of endogenously expressed CPSF6 to in vitro assembled HIV-1 CA-NC complexes.** (**A**) Cellular extracts from human 293T cells were incubated with wild type or mutant (N74D) in vitro assembled HIV-1 CA-NC complexes for 1 h. Samples were subsequently applied onto 70% sucrose cushion and centrifuged, as described in Methods. A small fraction of each lysate was collected before centrifugation and analyzed by Western Blotting using anti-CPSF6 antibodies (**INPUT**). Pelleted fractions (**BOUND**) were analyzed for the presence of CPSF6 and HIV-1capsid (p24) by Western blotting using anti-CPSF6 and anti-p24 antibodies, respectively. (**B**, **C**) The ability of endogenously expressed CPSF6 from human 293T cells to bind in vitro assembled HIV-1 CA-NC complexes was measured in the presence of the small molecule PF74 (PF-3450074). (**D**) The ability of the restriction factor TRIMCyp to bind in vitro assembled HIV-1 CA-NC in the presence of PF74. (**E**) Similarly, we measured the ability of endogenously expressed CPSF6 from TNPO3 K.D. and shRNA control HeLa cells to bind in vitro assembled HIV-1 CA-NC complexes. (**F**) HeLa cells stably expressing the nuclearly localized CPSF6 and the cytoplasmicly localized NES-CPSF6 were lysed in capsid binding buffer (10 mM Tris pH 7.4, 1.5 mM MgCl2, 10 mM KCl, 0.5 mM DTT) or whole cell extract buffer (50 mM Tris pH 8, 2 mM MgCl_2_, 280 mM NaCl, 0.5% NP-40,10% Glycerol). Extracts were analyzed by Western blotting using antibodies against FLAG. As a control, we blotted extracts using antibodies against the nuclear marker β-laminin. Similar results were obtained in three independent experiments and a representative experiment is shown.

Previous work has demonstrated the ability of CPSF6-358 fragment to bind in vitro assembled HIV-1 CA-NC complexes, suggesting that the CPSF6-358 fragment might be interacting with the incoming HIV-1 core [12,14]. We initially tested the ability of endogenously expressed CPSF6 to bind in vitro assembled wild type and mutant (N74D) HIV-1 capsid-nucleocapsid (CA-NC) complexes, as described [15]. In agreement with the infectivity phenotype, endogenously expressed CPSF6 in human 293 T cells only interacts with wild type HIV-1 CA-NC complexes (Figure 2A). To further test specificity of our binding system, we tested the ability of endogenously expressed CPSF6 to bind in vitro assembled HIV-1 CA-NC complexes in the presence of the HIV-1 small molecule inhibitor PF74 (PF-3450074) [16]. Interestingly, PF74 disrupts the binding of endogenously expressed full-length CPSF6 to HIV-1 CA-NC complexes demonstrating that the binding is specific (Figure 2B and C), in agreement with a previous report that studies the interaction of capsid with a 15-aminoacid peptide derived from CPSF6 in the presence of PF74 [17]. As a negative control, we measured the ability of PF74 to affect the binding of the restriction factor TRIMCyp to in vitro assembled HIV-1 CA-NC complexes (Figure 2D). Altogether, these experiments showed that the binding of CPSF6 to in vitro assembled HIV-1 CA-NC complexes is specific.

Because TNPO3-depleted HeLa cells require full-length CPSF6 for inhibition of HIV-1, we tested whether depletion of TNPO3 affects the ability of CPSF6 to bind HIV-1 capsid (Figure 2E). For this purpose, we measured the ability of endogenously expressed CPSF6 from TNPO3-depleted cells to bind in vitro assembled HIV-1 CA-NC complexes. As shown in Figure 2E, endogenously expressed CPSF6 from TNPO3-depleted cells bind in vitro assembled HIV-1 CA-NC complexes similar to CPSF6 from shRNA control cells. To show that extracts prepared using the capsid binding buffer registered changes in cytosolic content, we prepared extracts from HeLa cells stably expressing the nuclearly localize CPSF6 and the cytoplasmicly localized NES-CPSF6 (described below). As shown in Figure 2F, extracting with the capsid binding buffer showed more NES-CPSF6 when compared to CPSF6 suggesting that extracts prepared with the capsid binding buffer are able to register changes in cytoplasmic content. As a control, we extracted total protein using the whole cell extract buffer. Overall these experiments showed that depletion of TNPO3 in HeLa cells does not change the ability of CPSF6 to bind in vitro assembled HIV-1 CA-NC complexes.

### Depletion of TNPO3 does not change the localization of CPSF6 but it changes the localization of ASF/SF2

**Figure 3:**
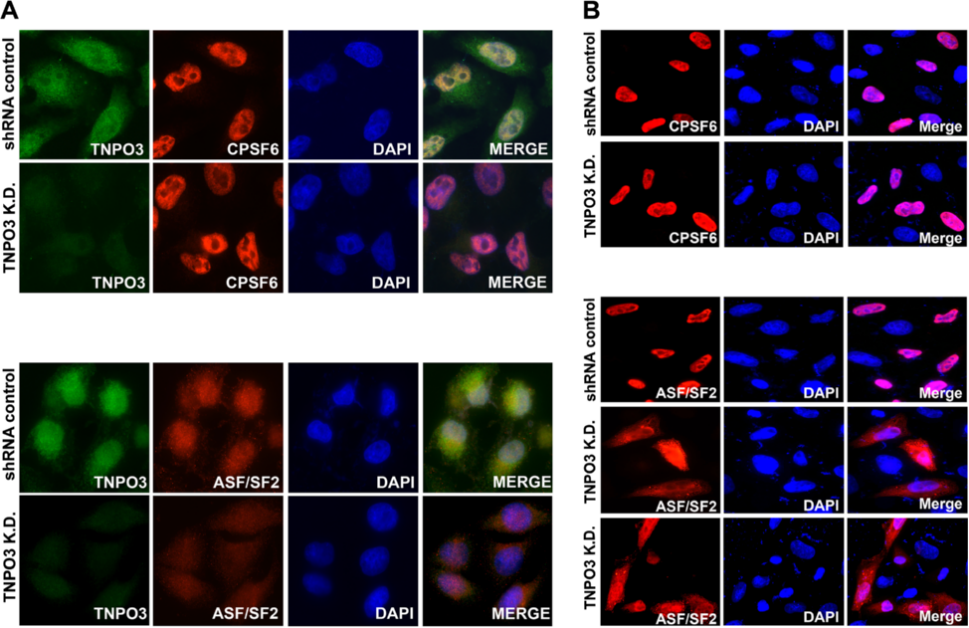
**Depletion of TNPO3 does not change the localization of CPSF6.** (**A**) TNPO3 K.D. and shRNA control HeLa cells were fixed and stained using specific antibodies against CPSF6 (red), ASF/SF2 (red) and TNPO3 (green), as described in Methods. The nuclear compartment was labeled using DAPI. Image quantification is shown in Additional file 1. (**B**) TNPO3 K.D. and shRNA control HeLa cells were transiently transfected using constructs expressing FLAG-tagged CPSF6 or ASF/SF2. Twenty-four hours post-transfection cells were fixed and immunostained using anti-FLAG antibodies. The nuclear compartment was labeled using DAPI. Image quantification is shown in Additional file 2. Similar results were obtained in three independent experiments and a representative experiment is shown.

Because CPSF6 is an SR protein, and TNPO3 is a nuclear import receptor for SR proteins, we tested whether depletion of TNPO3 changes the subcellular localization of CPSF6. As shown in Figure 3A, the subcellular distribution of CPSF6 remained nuclear in TNPO3 K.D. cells, as previously shown [1]. As a control, we studied the subcellular distribution of the SR protein alternative splicing factor/splicing factor 2 (ASF/SF2), which is known to interact with TNPO3 [18]. As shown in Figure 3A, the cytosol of TNPO3 K.D. cells showed an increase of ASF/SF2 when compared to shRNA control cells. Image quantification is shown in Additional file 1. Altogether these experiments showed that contrary to ASF/SF2, CPSF6 subcellular distribution does not change upon depletion of TNPO3 expression. These observations are also in agreement with our biochemical fractionation experiments where CPSF6 remained nuclear in TNPO3 K.D. cells [1].

To further confirm that TNPO3-depleted cells do not change the cellular distribution of CPSF6, we studied the cellular localization of transiently transfected CPSF6 or ASF/SF2 in TNPO3 K.D. cells (Figure 3B). For this purpose, we transfected CPSF6-FLAG or ASF/SF2-FLAG in TNPO3 K.D. cells, and study cellular distribution using anti-FLAG antibodies. In agreement, we found that CPSF6-FLAG did not change cellular distribution in TNPO3 K.D. cells when compared to transfection of shRNA control cells (Figure 3B). By contrast, ASF/SF2 mislocalized to the cytoplasm of TNPO3 K.D. cells when compared to transfections of shRNA control cells. Image quantification is shown in Additional file 2. Overall, our findings suggested that depletion of TNPO3 did not change the cellular distribution of CPSF6.

### Effect of TNPO3-depletion on the generation of 2-LTR circles during HIV-1 infection

**Figure 4:**
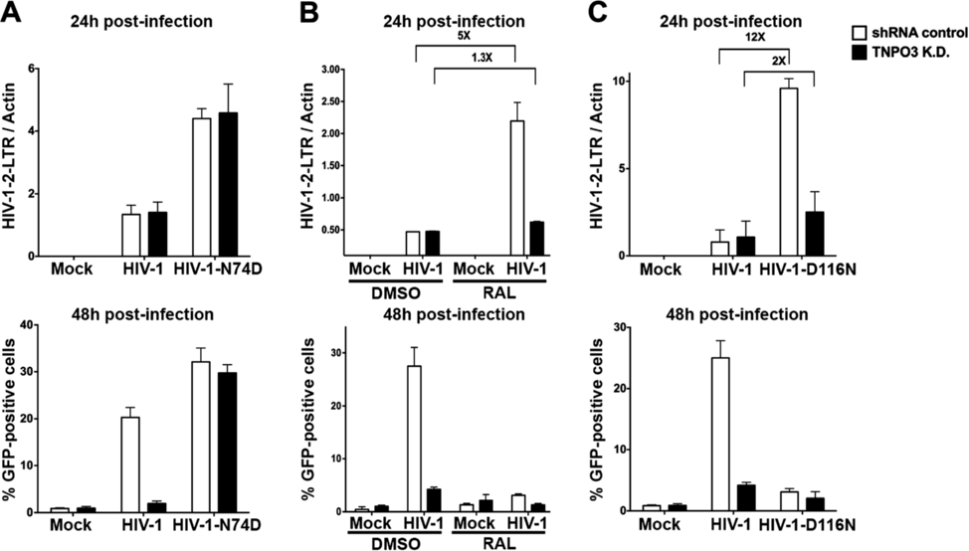
**TNPO3 K.D. cells block HIV-1 infection after nuclear import.** (**A**) TNPO3 K.D. and shRNA control HeLa cells were challenged with HIV-1 or HIV-1-N74D. Formation of 2-LTR circles and productive infection was measured twenty-four and forty-eight hours post-infection, respectively. Formation of 2-LTR circles was measured by real-time PCR. Productive infection was determined by measuring the percentage of GFP-positive cells by flow cytometry 48 hours post-infection. (**B**) Similarly, TNPO3 K.D. and shRNA control HeLa cells were challenged with HIV-1 in the presence of the HIV-1 integrase inhibitor raltegravir, and the formation of 2-LTR circles and productive infection was determined as described above. (**C**) Formation of 2-LTR circles and productive infection was also measured by infecting TNPO3 K.D. and shRNA control HeLa cells with HIV-1 and HIV-1-D116N, which is a virus that contain a defective integrase. Similar results were obtained in three independent experiments and standard deviations are shown. **Mock** refers to control cells that were not infected. Viruses were normalized by quantifying the particle-associated reverse transcriptase activity on viral supernatants, as described in Methods.

To explore the role of TNPO3 during HIV-1 infection, we challenged shRNA control and TNPO3 K.D. cells with HIV-1 and HIV-1-N74D to measure the formation of 2-LTR circles and productive infection 24 and 48 hours post-infection, respectively (Figure 4A). As previously shown depletion of TNPO3 blocks HIV-1 infection after nuclear import. Similarly, HIV-1-N74D was independent of TNPO3 (Figure 4A).

After completion of reverse transcription the viral DNA is translocated into the nucleus, where is integrated into the cellular genome by the HIV-1 integrase or ligated to form 2-LTR circles by nuclear ligases. To explore whether depletion of TNPO3 impact the occurrence of these processes, we performed infection of shRNA control and TNPO3 K.D. cells with HIV-1 in the presence of the integrase inhibitor raltegravir (RAL), and measure formation of 2-LTR circles and productive infection. As shown in Figure 4B, the use of RAL, which blocks the integration of viral DNA into the cellular genome, increases the amount of 2-LTR circles during HIV-1 infection (Figure 4B); the increase of 2-LTR circles during HIV-1 infection of shRNA control cells in the presence of raltegravir suggested that most of the viral DNA was routed to the formation of 2-LTR circles. Interestingly, we did not observe an increase of 2-LTR circles during HIV-1 infection of TNPO3 K.D. cells in the presence of raltegravir. To corroborate these findings, we challenged TNPO3 K.D. cells with an HIV-1 virus containing the integrase mutation D116N (HIV-1-D116N), which results in a defective integrase [19,20]. Similarly, HIV-1-D116N infection of TNPO3 K.D. cells did not exhibited an increase in the levels of 2-LTR circles when compared to the infection of wild type HIV-1 (Figure 4C). Overall these results suggested that TNPO3-depleted cells are impaired in the integration process or exhibit a defect in the formation of 2-LTR circles.

### Expression of a cytosolic full-length CPSF6

**Figure 5:**
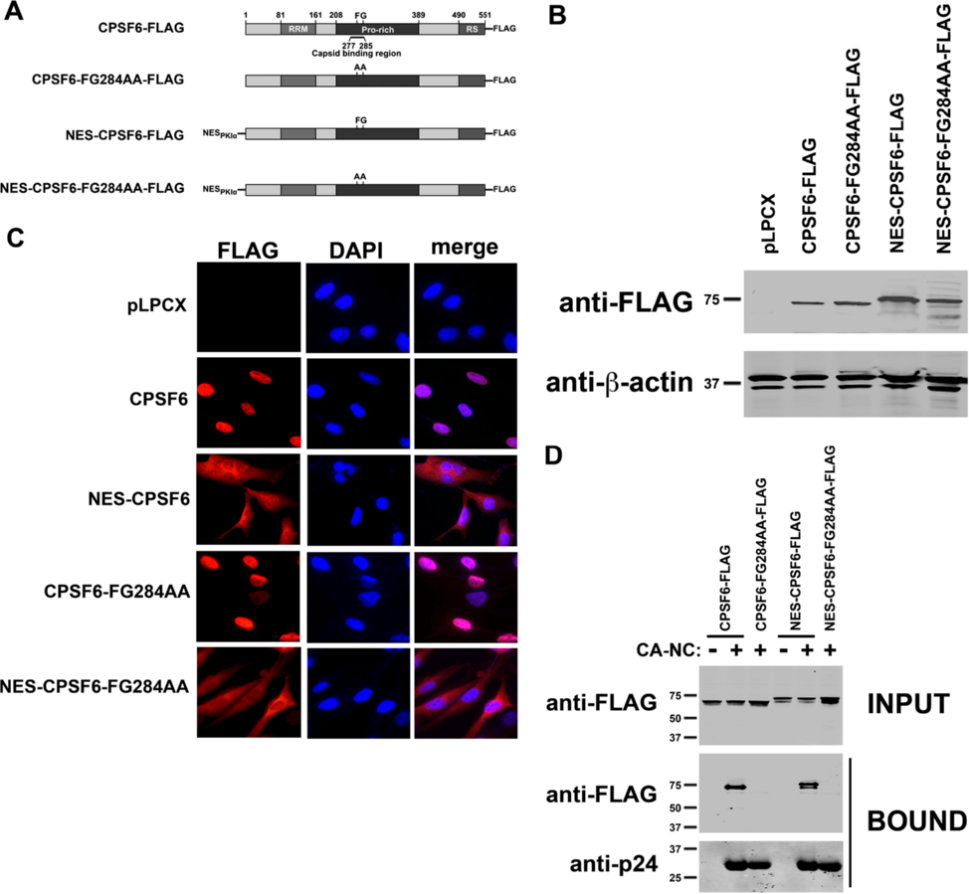
**Expression of a cytosolic full-length CPSF6.** (**A**) The wild type CPSF6 (NCBI Reference Sequence: NP_008938.2) protein with a C-terminal FLAG epitope is depicted on top. The numbers of the amino acid residues at the boundaries of the different domains are indicated (RRM: RNA recognition motive, Pro-rich: Proline-rich domain, RS: Arginine/Serine repeats). The HIV-1 capsid binding region is shown (residues 277-285). The nuclear export signal of the protein kinase inhibitor α (NES-PKIα). The amino acid sequence of NES-PKIα is NELALKLAGLDI. The NES-PKIα was fused to the N-terminus of CPSF6. (**B**) Cf2Th cells stably transduced with the different CPSF6 variants were analyzed for expression by Western blotting using anti-FLAG antibodies. As a loading control, cell lysates were Western blotted against β-actin. (**C**) Intracellular distribution of the different CPSF6 variants stably expressed in Cf2Th was studied by immunofluorescence microscopy, as described in Methods. The different CPSF6 variants were stained using anti-FLAG antibodies (red). The cellular nuclei were stained by using DAPI (blue). Image quantification is shown in Additional file 3. (**D**) The ability of the different CPSF6 variants to bind in vitro assembled HIV-1 CA-NC complexes was measured. 293T cells were transfected with plasmids expressing the indicated CPSF6 variants. Thirty-six hours after transfection, cells were lysed. The lysates were incubated at room temperature for 1hour with in vitro assembled HIV-1 CA-NC complexes. The mixtures were applied to a 70% sucrose cushion and centrifuged. **INPUT** represents the lysates analyzed by Western blotting before being applied to the 70% sucrose cushion. The input mixtures were Western blotted using anti-FLAG antibodies. The pellet from the 70% sucrose cushion (**BOUND**) was analyzed by Western blotting using anti-FLAG and anti-p24.

We have demonstrated that inhibition of HIV-1 infection by TNPO3-depleted cells require full-length CPSF6. To understand whether inhibition of HIV-1 by TNPO3 K.D. cells is due to the CPSF6 present in the cytoplasm, we created a CPSF6 protein that localizes to the cytoplasm. For this purpose, we fused the full-length CPSF6 protein to the nuclear export signal of the protein kinase inhibitor alpha (NES-CPSF6) [21] (Figure 5A). As a control, we mutated the HIV-1 capsid binding region of CPSF6 (NES-CPSF6-FG284AA) (Figure 5A) [14]. We stably transduced canine Cf2Th cells with the indicated CPSF6 variants and measure protein expression by Western blot using antibodies against FLAG (Figure 5B). To test whether the NES changes the localization of CPSF6, we studied the subcellular localization of the different CPSF6 variants by immunofluorescence microscopy. Contrary to the full-length CPSF6 protein that exclusively localizes to the nucleus, the NES-CPSF6 variants localized to the nucleus and the cytoplasm (Figure 5C). Similarly, the NES-CPSF6-FG284AA also localized to the nucleus and cytoplasm. Image quantification for Figure 5C is shown in Additional file 3.

Next, we tested the ability of the different CPSF6 variants to bind in vitro assembled HIV-1 CA-NC complexes. As shown in Figure 5D, NES-CPSF6 bound in vitro assembled HIV-1 CA-NC complexes as strong as the wild type CPSF6. Interestingly, variants containing mutations in the capsid binding region lost the ability to bind in vitro assembled HIV-1 CA-NC complexes when compared to wild type CPSF6 (Figure 5D). These results showed the establishment of a cytosolic CPSF6.

### Cytosolic CPSF6 restricts HIV-1 replication after reverse transcription but before or at nuclear import

**Figure 6:**
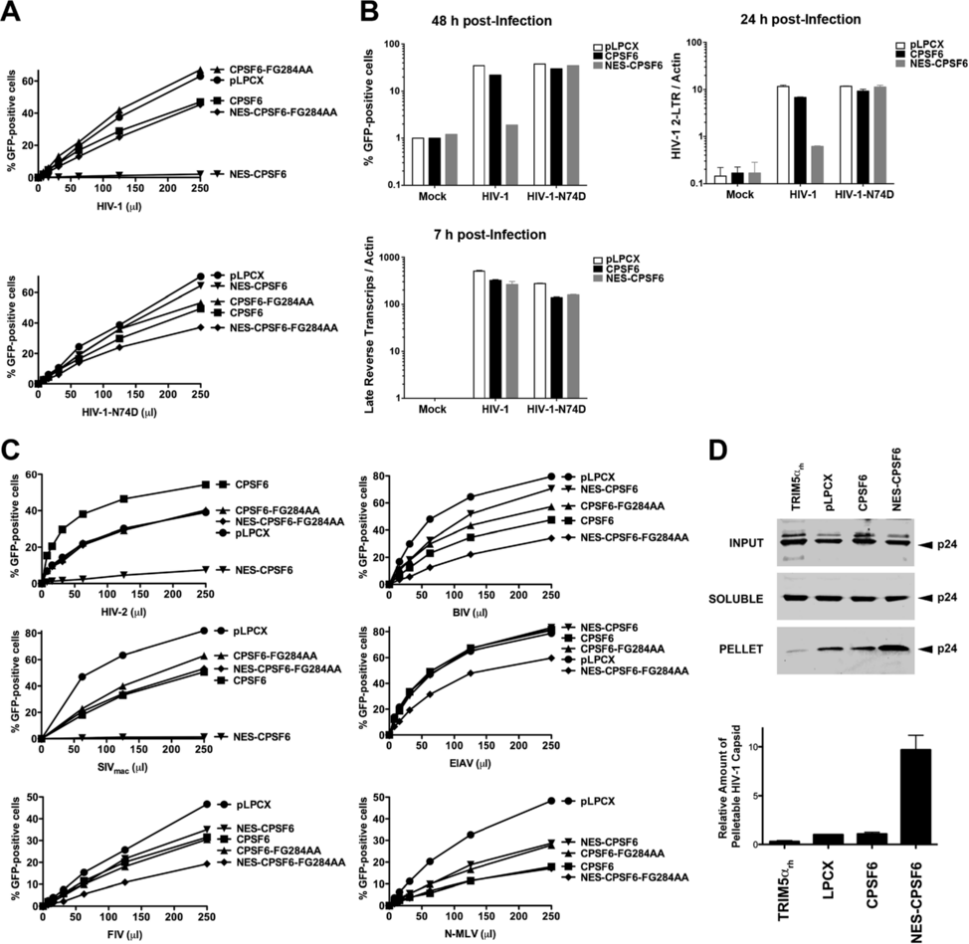
**Cytosolic CPSF6 expressed in Cf2Th cells restricts HIV-1 replication before or at the nuclear import step.** (**A**) Cf2Th cells stably expressing the indicated CPSF6 constructs were challenged with HIV-1 or HIV-1-N74D expressing GFP as a reporter. Forty-eight hours post-infection the percentage of GFP-positive cells was determined by flow cytometry. As control, Cf2Th cells stably transduced with the empty vector pLPCX were challenged with HIV-1 and HIV-1-N74D. (**B**) Cf2Th cells stably expressing CPSF6 and NES-CPSF6 were challenged with the indicted viruses. Infection was determined by measuring the percentage of GFP-positive cells by flow cytometry 48 hours post-infection (upper panel). In parallel, cells from similar infections were lysed at 7 or 24 hours post-infection and total DNA was extracted. The DNA samples collected at 7 hours post-infection were used to determine the levels of late reverse transcripts by real-time PCR (middle panel). Separately, DNA samples collected at 24 hours post-infection were used to quantify HIV-1 2-LTR circles by real-time PCR in DNA (lower panel). **Mock** refers to control cells that were not infected. (**C**) Cf2Th cells stably expressing the different CPSF6 variants were challenged with HIV-2, SIVmac, FIV, BIV, EIAV or N-MLV. As control, Cf2Th cells stably transduced with the empty vector pLPCX were challenged with HIV-2, SIVmac, FIV, BIV, EIAV or N-MLV. Infectivity by the different viruses was determined by measuring the percentage of GFP-positive 48 hours post-infection. (**D**) Cf2Th cells stably expressing the indicated CPSF6 variant or TRIM5α_rh_ were challenged using similar amounts of HIV-1, and performed the fate of the capsid assay to separate pelletable from soluble cytosolic capsids 16 hours post-infection as described in Methods. Input, soluble and pellet fractions were analyzed by Western blotting using antibodies against HIV-1 CA p24. Similar results were obtained in three independent experiments and a representative experiments is shown.

We tested the ability of NES-CPSF6 to block HIV-1 infection. For this purpose, we challenged Cf2Th cells stably expressing the different CPSF6 variants by increasing amounts of HIV-1 expressing GFP as a reporter for infection. Contrary to CPSF6, the NES-CPSF6 variant potently block HIV-1 infection (Figure 6A). In agreement with our capsid binding assays, the NES-CPSF6-FG284AA variant that contains a mutation in the capsid binding region did not block HIV-1 infection (Figure 6A). As a control, we performed similar infections using HIV-1-N74D, which is insensitive to the block imposed by CPSF6-358 [12]. These results showed that expression of a cytosolic full-length CPSF6 potently blocks HIV-1 infection.

To understand whether the inhibition of HIV-1 imposed by TNPO3-depleted cells is similar to the block imposed by a cytosolic CPSF6, we investigated the viral stage at which NES-CPSF6 blocks HIV-1. For this purpose, we challenged Cf2Th cells stably expressing NES-CPSF6 by HIV-1-GFP and measure the percentage of GFP-positive cells (Figure 6B). Similar infections were performed to monitor late reverse transcripts 7 hours post-infection, and the formation of 2-LTR circles 24 hours post-infection (Figure 6B). These results showed that NES-CPSF6 blocks HIV-1 infection at the level of nuclear import (Figure 6B), while HIV-1 inhibition by TNPO3 K.D. HeLa cells occurs after nuclear import.

Next we tested the ability of cytosolic CPSF6 to restrict different viruses, including HIV-2, simian immunodeficiency virus from macaques (SIVmac), feline immunodeficiency virus (FIV), bovine immunodeficiency virus (BIV), equine infectious anemia virus (EIAV), and N-tropic murine leukemia virus (N-MLV). In agreement with previous observations [12], cytosolic CPSF6 inhibited infection of HIV-2 and SIVmac (Figure 6C).

Because CPSF6 binds the HIV-1 core, we decided to test whether the cytoplasmic CPSF6 (NES-CPSF6) influences the stability of the HIV-1 core during infection. For this purpose, we infected Cf2Th cells stably expressing NES-CPSF6 or CPSF6 using similar amounts of HIV-1, and performed the fate of the capsid assay 16 hours post-infection as previously described [22]. Interestingly, NES-CPSF6 increased the stability of the HIV-1 core during infection by at least 10 fold (Figure 6D). As a control, we observed the ability of TRIM5α_rh_ to accelerate the uncoating process of HIV-1 (Figure 6D). These results suggested that NES-CPSF6 stabilizes the HIV-1 core during infection preventing productive infection.

**Figure 7:**
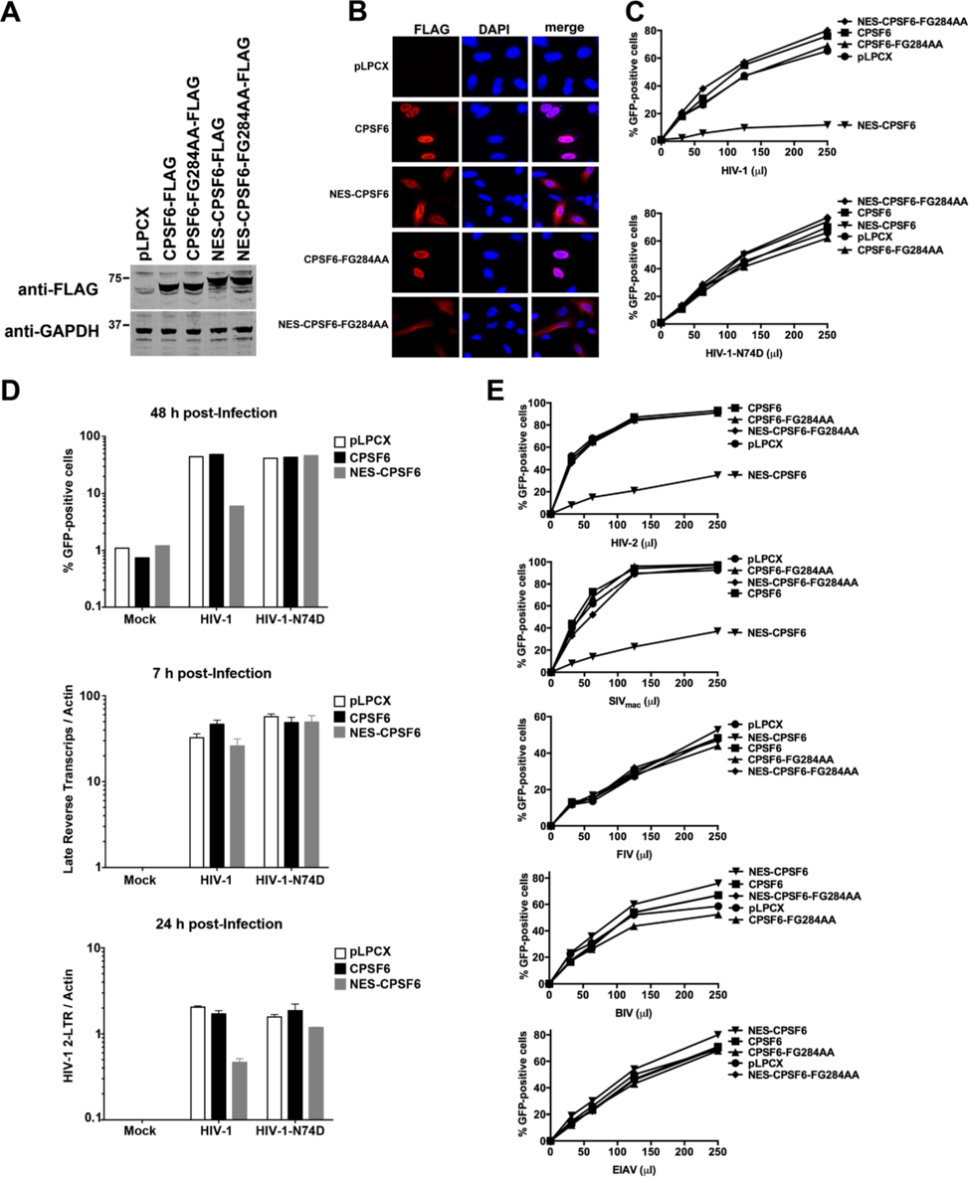
**Cytosolic CPSF6 expressed in human HeLa cells restricts HIV-1 replication before or at the nuclear import step.** (**A**) HeLa cells were stably transduced with the indicated CPSF6 variant. Expression of the different CPSF6 variants was analyzed by Western blotting using anti-FLAG antibodies. (**B**) The cellular distribution of the different CPSF6 variants was studied by immunofluorescence using anti-FLAG antibodies (red). The nuclear compartment was stained using DAPI (blue). Image quantification is shown in Additional file 4. (**C**) HeLa cells stably expressing the indicated CPSF6 variants were challenged with HIV-1 or HIV-1-N74D expressing GFP as a reporter for infection. Forty-eight hours post-infection the percentage of GFP-positive cells was determined by flow cytometry. As control, HeLa cells stably transduced with the empty vector pLPCX were challenged with increasing amounts of HIV-1 and HIV-1-N74D. (**D**) HeLa cells stably expressing CPSF6 and NES-CPSF6 were challenged with the indicted viruses. Infection was determined by measuring the percentage of GFP-positive cells by flow cytometry 48 hours post-infection (upper panel). In parallel, similarly infected cells were lysed at 7 or 24 hours post-infection and total DNA was extracted. The DNA samples collected at 7 hours post-infection were used to determine the levels of late reverse transcripts by real-time PCR (middle panel). DNA samples collected at 24 hours post-infection were used to quantify HIV-1 2-LTR circles by real-time PCR (lower panel). **Mock** refers to control cells that were not infected. (**E**) HeLa cells expressing the different CPSF6 variants were challenged with HIV-2, SIVmac, FIV, BIV or EIAV. As control, HeLa cells stably transduced with the empty vector pLPCX were challenged with HIV-2, SIVmac, FIV, BIV or EIAV. Infectivity by the different viruses was determined by measuring the percentage of GFP-positive 48 hours post-infection. Similar results were obtained in three independent experiments and a representative experiments is shown.

We performed similar experiments in the human cell line HeLa. For this purpose, we stably expressed the different CPSF6 variants in human HeLa cells (Figure 7A). In agreement NES-CPSF6 localized to the cytoplasm of HeLa cells (Figure 7B). Image quantification is shown in Additional file 4. NES-CPSF6 expressed in HeLa cells blocked infection by HIV-1 but not HIV-1-N74D (Figure 7C). Similar to our findings in Cf2Th cells, NES-CPSF6 expressed in HeLa cells blocked HIV-1 infection at the level of nuclear import (Figure 7D). In addition, we found that NES-CPSF6 expressed in HeLa cells also blocked infection of HIV-2 and SIVmac (Figure 7E).

## Discussion

This work studied the role of CPSF6 in the inhibition of HIV-1 by TNPO3-depleted cells. Our siRNA depletion experiments showed that CPSF6 is necessary for the ability of TNPO3-depleted cells to inhibit HIV-1 infection. The contribution of CPSF6 to the ability of TNPO3-depleted cells to inhibit HIV-1 infection might be due to a direct effect of CPSF6 on HIV-1 infection. We favor three different mechanisms by which CPSF6 might be necessary for HIV-1 inhibition in TNPO3-depleted cells: 1) CPSF6 directly binds a viral component in the cytoplasm, 2) CPSF6 interacts with a viral component in the nucleus, or 3) CPSF6 interacts with a component of the virus in the cytoplasm and in the nucleus. To distinguish among these models, we studied the HIV-1 capsid binding ability and the subcellular localization of endogenous CPSF6 in TNPO3-depleted cells. The ability of CPSF6 from TNPO3-depleted cells to bind in vitro assembled HIV-1 CA-NC components did not change when compared to CPSF6 from control cells. These experiments showed that the ability of CPSF6 to bind the HIV-1 core is not affected in TNPO3-depleted cell. Subcellular localization experiments showed that the predominant nuclear localization of full-length CPSF6 is unaffected in TNPO3-depleted cells, which is in agreement with our biochemical fractionation experiments [1]. The unchanged nuclear localization of CPSF6 suggested that the effect of CPSF6 on the virus occurs in the nucleus. Alternatively, our detection methods might not be sensitive enough to detect subcellular distribution changes.

To further understand the role of CPSF6 in the inhibition of HIV-1 observed in TNPO3-depleted cells, we measured the levels of 2-LTR circles during infection of TNPO3 K.D. cells. In agreement with previous observations [1-3,11], we found that TNPO3-depleted cells inhibit HIV-1 infection after nuclear import, as suggested by the presence of 2-LTR circles. The use of raltegravir or the HIV-1 mutant defective on Integrase D116N in TNPO3 K.D. cells suggested that these cells might be affecting integration or the formation of 2-LTR circles. One possibility is that TNPO3 is important for a step in the maturation process of the pre-integration complex, which is essential for the integration to occur. Because TNPO3 also binds capsid [1,13], an interesting possibility is that TNPO3 is recruited to the pre-integration complex by the capsid in order to allow this maturation step to occur. Overall, this evidence contributes to the notion that TNPO3-depleted cells might be affecting the maturation process of the pre-integration complex, which could result in an HIV-1 integration defect, as previously suggested [13].

Because overexpression of a CPSF6 fragment, CPSF6-358 [12], potently blocks HIV-1 infection, we tested whether overexpression of a full-length CPSF6 protein inhibits HIV-1 infection. Differently from TNPO3-depleted cells, overexpression of cytosolic full-length CPSF6 inhibited HIV-1 infection before nuclear import. These experiments suggested that the observed inhibition of HIV-1 by TNPO3-depleted cells is different from the inhibition caused by overexpressing a full-length cytosolic CPSF6. One possibility is that the stage of the block is related to the expression levels of CPSF6 in the cytosol. For example, at the endogenous levels of CPSF6 the block in TNPO3-depleted cells is observed after nuclear import. However, when CPSF6 is overexpressed in the cytosol of wild-type HeLa cells the block is before or at nuclear import. Future experiments will attempt to understand more mechanistically the role of CPSF6 in the observed HIV-1 inhibition in TNPO3-depleted cells.

## Conclusions

These results suggested that inhibition of HIV-1 by TNPO3-depleted cells requires CPSF6.

## Methods

### Cell lines and plasmids

Human 293T cells, human HeLa cells and dog Cf2Th cells were grown on DMEM supplemented with 10% fetal bovine serum and 1% (w/v) penicillin/streptomycin. HeLa cells: TNPO3 K.D and shRNA control were constructed as previously described [1]. CPSF6 (NCBI Reference Sequence: NP_008938.2) fused to C-terminal FLAG epitope was cloned in the pLPCX vector (Clontech) using EcoRI site. The nuclear export signal of protein kinase inhibitor α (amino acid sequence: NELALKLAGLDI) was inserted on the N-terminus of CPSF6 by PCR-cloning. Plasmids expressing the CPSF6 capsid binding mutants (FG284AA) were created by site directed mutagenesis. Constructs were confirmed by sequencing analysis.

### Generation of Cf2Th cells stably expressing CPSF6 variants

Cf2Th cells were stably transduced with recombinant retroviruses containing the different CPSF6 variants. Recombinant viruses were produced in 293T cells by co-transfecting the LPCX plasmids with the pVPack-GP and pVPack-VSV-G packaging plasmids (Stratagene). The pVPack-VSV-G plasmid encodes the vesicular stomatitis virus G envelope glycoprotein, which allows efficient entry into a wide range of vertebrate cells [23]. Transduced dog Cf2Th cells were selected in 4 μg/ml puromycin (Sigma).

### Western blot analysis

Cellular proteins were extracted with radioimmunoprecipitation assay (RIPA) as previously described [15]. Detection of proteins by Western blotting was performed using anti-FLAG (Sigma), anti-CPSF6 (abcam), anti-GAPDH (Ambion), anti-β-Actin (Sigma) or anti-p24 (Immuno Diagnostics) antibodies. Secondary antibodies against rabbit and mouse conjugated to IRDye 680LT or IRDye 800CW were obtained from LI-COR. Bands were detected by scanning blots using the LI-COR Odyssey Imaging System in the 700 nm or 800 nm channel.

### Infection with retroviruses expressing the green fluorescent protein (GFP)

Recombinant retroviruses expressing GFP, pseudotyped with the VSV-G glycoprotein, were prepared as described [24]. For infections, 6 × 10^4^ cells seeded in 24-well plates were incubated with the indicated retrovirus for 48 hours at 37°C. The percentage of GFP-positive cells was determined by flow cytometry (Becton Dickinson). Viral stocks were titrated by serial dilution on dog Cf2Th cells.

### HIV-1 CA-NC expression and purification

The HIV-1 CA-NC protein was expressed, purified and assembled as previously described [15]. The pET11a expression vector (Novagen) expressing the CA-NC protein of HIV-1 was used to transform BL-21(DE3) E. coli. CA-NC expression was induced with 1 mM isopropyl-β-D-thiogalactopyranoside (IPTG) when the culture reached an optical density of 0.6 at 600 nm. After 4 hours of induction, the cells were harvested and resuspended in 20 mM Tris-HCl (pH 7.5), 1 μM ZnCl_2_, 10 mM 2-mercaptoethanol and protease inhibitors (Roche). Lysis was performed by sonication, and debris were pelleted for 30 minutes at 35,000×g. Nucleic acids were stripped from the solution by using 0.11 equivalents of 2M (NH_4_)_2_SO_4_ and the same volume of 10% polyethylenimine. Nucleic acids were removed by stirring and centrifugation at 29,500×g for 15 minutes. The protein was recovered by addition of 0.35 equivalents of saturated (NH_4_)_2_SO_4_. The protein was centrifuged at 9,820×g for 15 minutes and resuspended in 100 mM NaCl, 20 mM Tris-HCl (pH 7.5), 1 μM ZnCl_2_ and 10 mM 2-mercaptoethanol. Lastly the CA-NC protein was dialyzed against 50 mM NaCl, 20 mM Tris-HCl (pH 7.5), 1 μM ZnCl_2_ and 10 mM 2-mercaptoethanol, and stored at -80°C.

### In vitro assembly of wild type and N74D HIV-1 CA-NC complexes

HIV-1 CA-NC particles were assembled in vitro by diluting the CA-NC protein to a concentration of 0.3 mM in 50 mM Tris-HCl (pH 8.0), 0.5 M NaCl and 2 mg/ml DNA oligo-(TG)50. The mixture was incubated at 4°C overnight and centrifuged at 8,600×g for 5 minutes. The pellet was resuspended in assembly buffer (50 mM Tris-HCl (pH 8.0), 0.5 M NaCl) at a final protein concentration of 0.15 mM, and stored at 4°C until needed.

### Binding of CPSF6 to in vitro assembled HIV-1 CA-NC complexes

To study the binding of endogenous CPSF6 to in vitro assembled HIV-1 CA-NC complexes, we lysed 2x10^6^ HeLa or 293T cells in capsid binding buffer (10 mM Tris-HCl pH 7.4, 1.5 mM MgCl2, 10 mM KCl, 0.5 mM DTT) and incubated for 15 min at 4°C. The lysates were centrifuged in a refrigerated Eppendorf microcentrifuge (~14,000×*g*) for 15 minutes. To test the binding of the different CPSF6 variants, human 293T cells were transfected with the different CPSF6 plasmids. 400 μl of cell lysates were incubated with 5 μl of in vitro assembled HIV-1 CA-NC complexes and incubated at room temperature for 1 hour. A portion of this mixture, henceforth referred to as “INPUT” was stored. The mixture was spun through a 70% sucrose cushion (70% sucrose, 1X PBS and 0.5 mM DTT) at 100,000×*g* in an SW55 rotor (Beckman) for 1h at 4°C. After centrifugation, the supernatant was carefully removed and the pellet was resuspended in 1X SDS-PAGE loading buffer and henceforth referred to as (BOUND). The level of CPSF6 proteins was determined by Western blotting using anti-CPSF6 or anti-FLAG antibodies. The level of HIV-1 CA-NC protein in the pellet (BOUND) was assessed by Western blotting using anti-p24 antibodies.

### siRNA transfection

siRNA smart pool targeting CPSF6 (Dharmacon, ON-TARGETplus SMARTpool L-012334-01-005; targeting Human CPSF6, NCBI Reference Sequence: NM_007007) was used to transiently silence CPSF6 expression in TNPO3 K.D. and shRNA control HeLa cells. All siRNA experiments were performed with oligofectamine (Invitrogen, 12252-011) according to manufacturer’s protocol with no modification. DMEM without serum was used instead of Opti-MEM medium. The amount of siRNA smart pool to efficiently silence CPSF6 was 100 nM. Forty-eight hours post-transfection, cells were challenged with increasing amounts of the indicated HIV-1 viruses. Infections were incubated for 48 hours, and infectivity was determined by measure the percentage of GFP-positive cells using a flow cytometer.

### Quantification of particle associated reverse transcriptase activity

Particle-associated reverse transcriptase was measured as previously described [25]. 1 ml of virus was divided into 3 separate samples and spun at 12000 rpm, 4ºC for 1h. The pellet was resuspended by vortexing in 12 μl of RT suspension buffer (50 mM Tris-HCL pH 7.5, 1mM DTT, 20% Glycerol, 250 mM KCl, 0.25% Triton X-100). Each sample was Freeze-thaw and vortexed 3 times. 50 μl of RT assay reaction mix (50 mM Tris-HCl pH 7.5, 7.5 mM MgCl_2_, 0.05% Triton X-100, 5.5 mM DTT, 0.6 u/ml Poly[A] p[dT]_10_ (Sigma, p-4797) ,24 μCi/ml ^3^H-dTTP) was added to each sample and it was incubate at 37ºC for 1h. The samples were pipetted into a small Whatman filter (DE81), soaked in SSC buffer (30 mM Na-citrate-HCl pH 7, 250 mM NaCl), washed 3 times for 10 min in SSC buffer and washed 2 times for 10 sec in 95% Ethanol. The filter was dried and the radioactivity of ^3^H was measured using a Scintillation Counter (Perkin Elmer).

### Transfections and immunofluorescence microscopy of FLAG-tagged proteins

Transfections of cell monolayers were performed using Lipofectamine Plus reagent (Invitrogen), according to the manufacturer’s instructions. Transfections were incubated at 37ºC for 24 h. Indirect immunofluorescence microscopy was performed as previously described [26]. Transfected monolayers grown on coverslips were washed twice with PBS and fixed for 15 min in 3.9% paraformaldehyde in PBS. Fixed cells were washed twice in PBS, permeabilized for 4 min in permeabilizing buffer (0.5% TritonX-100 in PBS), and then blocked in PBS containing 2% bovine serum albumin (blocking buffer) for 1 h at room temperature. Cells were then incubated for 1 h at RT with primary antibodies diluted in blocking buffer. After three washes with PBS, cells were incubated for 30 min in secondary antibodies and 1 mg/ml of DAPI (4′,6-diamidino-2-phenylindole). Samples were mounted for fluorescence microscopy by using the Pro Long Antifade Kit (Molecular Probes, Eugene, OR). Images were obtained with a Zeiss Observer Z1 microscope using a 63X objective, and deconvolution was performed using the software AxioVisionV4.8.1.0 (Carl Zeiss Imaging Solutions).

### Immunofluorescence microscopy of endogenously expressed TNPO3, CPSF6 and ASF/SF2 in HeLa cells

4x10^4^ TNPO3 K.D. or shRNA control HeLa cells were seeded on 12-mm-diameter glass discs (Fisher Scientific) and incubated at 37ºC overnight. Cells were fixed in 4% paraformaldehyde (Electron Microscopy Sciences, cat.15710) in PBS for 30 minutes, washed twice in PBS. Samples were the incubated in 0.1 M glycine dissolved in PBS (Sigma) two times for 5 minutes and washed in PBS. Cells were incubated in permeabilization and blocking buffer (0.1% Triton-X 100 and 5% normal goat serum and 0.2% BSA) for 30 minutes. Cells were incubated overnight with primary antibodies in permeabilization and blocking buffer. The TNPO3 protein was stained using rabbit (Abcam cat# ab65016) or a mouse (cat# ab54353) anti-TNPO3 antibody. The CPSF6 protein was stained using a rabbit anti-CPSF6 antibody (Novus Biological cat# NB100-61596). The ASF/SF2 protein was stained using a mouse anti-ASF/SF2 antibody (Santa Cruz Biotechnology cat# cs-73026). Cells were washed three times in permeabilization and blocking buffer. Secondary antibodies were applied at a dilution of 1:1000 (anti-rabbit Cy3-conjugated antibodies, Jackson ImmunoResearch, cat#711-165-152 and anti- mouse Cy3-conjugated antibodies, Jackson ImmunoResearch, cat#715-165-151) or 1:500 (anti-mouse Alexa Fluor488-conjugated antibodies, Invitrogen, cat# A-11001 and anti-rabbit Alexa Fluor488-conjugated antibodies, Invitrogen, A-11094) for 1h and washed with PBS. Cells were stained with DAPI for 3-5min, following 3 washes with PBS. Subsequently, samples were mounted for fluorescent microscopy by using the FluorSave reagent (Calbiochem, cat. 345789). Images were obtained with a Zeiss Observer.Z1 microscope using a 63X objective. Images were deconvolved by using a defined point spread function in the software AxioVision V4.8.1.0 (Carl Zeiss Imaging Solutions).

### Quantitative real-time PCR for detection of HIV-1 and HIV-1-N74D late reverse transcripts (LRT) and 2-LTR circles

1x10^6^ cells were seeded in 10cm plates and challenged by Dpn1-pretreated viruses, including HIV-1, HIV-1-N74D or HIV-1-N116D. As control, we used heat inactivated viruses for 30 min at 100˚C. Total cellular DNA was harvested 7 hours post-infection to measure late reverse transcripts by real-time PCR. Similar infections were harvested 24 hours after infection for the measurement of 2-LTR circles by real-time PCR. Total cellular DNA was extracted by using the QIAamp DNA mini kit (QIAGEN). For the specific detection of late reverse transcript by real-time PCR, we used the following primers and probe: 5′GACGTAAACGGCCACAAG3′, 5′GGTCTTGTAGTTGCCGTCGT3′ and 5′/56-FAM/CCTACGGCAAGCTGACCC /36-TAMSp/-3. A standard curve was created using the GFP sequence of the HIV-1 reporter virus. Similarly, for the detection of 2-LTR circles, we used the following primers and probe: 5′ACCTAGGGAACCCACTGCTTAAG3′, 5′ TCC ACAGATCAAGGATATCTTGTC3′ and probe 5-/56-FAM/ACACTACTTGAAGCA CTCAAGGCAAGCTTT/36-TAMSp/-3. A standard curve was created using the pUC2LTR plasmid, which contains the HIV-1 2-LTR junction.

DNA samples for real-time PCR from human cells were normalized by amplification of the β-actin housekeeping gene using the following primers using SYBR Green(cat# 4309155): 5′ AACACCCCAGCCATGTACGT3′ and 5′ CGGTGAGGATCTTCATGAGGTAGT 3′. DNA samples for real-time PCR from canine Cf2Th cells were normalized by amplification of canine actin housekeeping gene using the following primers by SYBR Green: 5′GCATCCTGACCCTCAAGTAG 3′ and 5′ACATACATGGCTGCTGGGGTGTT3′.

### Fate of the capsid assay

The fate of the capsid assay was performed as previously described [27]. HIV-1 virus-like particles (VLPs) were produced by calcium phosphate co-transfection of plasmids containing the following genes: HIV-1 gag-pol, VSV-G envelope and rev protein at a weight:ratio 15:3:1. Stably transduced Cf2Th (1.5 × 10^6^) cells expressing the indicated proteins were seeded in 80 cm^2^ flasks. The following day, the cells were incubated with 5-10 ml (approximately 2.5- 5.0 × 10^5^ reverse transcriptase units) of HIV-1 at 4°C for 30 minutes to allow viral attachment to the cells. The cells were then shifted to 37°C until they were harvested 16 hours post-infection. Cells were washed three times using ice-cold PBS and detached by treatment with 1.0 ml of pronase (7.0 mg/ml in DMEM) for 5 minutes at 25°C. The cells were then washed three times with PBS. The cells were resuspended in 2.5 ml hypotonic lysis buffer (10 mM Tris-HCl, pH 8.0, 10 mM KCl, 1 mM EDTA and one complete protease inhibitor tablet) and incubated on ice for 15 minutes. The cells were lysed using 15 strokes in a 7.0 ml Dounce homogenizer with pestle B. Cellular debris were cleared by centrifugation for 3 minutes at 3000 rpm. To allow assessment of the INPUT for HIV-1 p24, 100 μl of the cleared lysate were collected, made 1x in SDS sample buffer, and analyzed by Western blotting. Then 2.0 ml of the cleared lysate were layered onto a 50% sucrose (weight:volume) cushion in 1x PBS and centrifuged at 125,000 x g for 2 hours at 4°C in a Beckman SW41 rotor. Following centrifugation, 100 μl of the top-most portion of the supernatant were collected and made 1x in SDS sample buffer; this sample is referred to as the SOLUBLE p24 (HIV-1 CA) fraction. The PELLET was resuspended in 50 μl 1x SDS sample buffer and is referred to as the particulate p24. All samples were then subjected to SDS-PAGE and Western blotting. The HIV-1 p24 proteins were detected using a mouse anti-p24 antibody (Immunodiagnostics).

## Competing interests

The authors declare that they have no competing interests.

## Authors’ contributions

TF performed experiments and helped with revision of the manuscript. JVC performed experiments and helped with revision of the manuscript. TEW performed experiments. ABN performed experiments. WB performed experiments. NR performed experiments. RG design experiments. FDG design experiments, wrote manuscript, and supervised the project. All authors read and approved the final manuscript.

## Supplementary Material

Additional file 1 Subcellular localization of CPSF6 in the different cell lines. Click here for file

Additional file 2 Subcellular localization of CPSF6 transfected in the different cell lines. Click here for file

Additional file 3 Subcellular localization of CPSF6. Click here for file

Additional file 4 Subcellular localization of CPSF6. Click here for file
