# Supplementary material for: The ability of TNPO3-depleted cells to inhibit HIV-1 infection requires CPSF6
Source: Retrovirology. 2013 Apr 26;10:46. doi: 10.1186/1742-4690-10-46 (PMC3695788; doi:10.1186/1742-4690-10-46)
Supplement: Additional file 1 — Subcellular localization of CPSF6 in the different cell lines. [file 1742-4690-10-46-S1.pdf]

	Subcellular localization of CPSF6 in the different cell lines.								
	Experiment 1			Experiment 2			Experiment 3		
HeLa cells	Exclusively nuclear	Exclusively cytoplasm	Throughout cell	Exclusively nuclear	Exclusively cytoplasm	Throughout cell	Exclusively nuclear	Exclusively cytoplasm	Throughout cell
shRNA control	198	0	2	184	0	16	189	0	11
TNPO3 K.D.	175	0	25	190	0	10	166	0	34

	Subcellular localization of ASF/SF2 in the different cell lines.								
	Experiment 1			Experiment 2			Experiment 3		
HeLa cells	Exclusively nuclear	Exclusively cytoplasm	Throughout cell	Exclusively nuclear	Exclusively cytoplasm	Throughout cell	Exclusively nuclear	Exclusively cytoplasm	Throughout cell
shRNA control	183	0	17	165	0	35	191	0	9
TNPO3 K.D.	35	0	165	55	0	145	43	0	157

AF 1
