# Supplementary material for: The ability of TNPO3-depleted cells to inhibit HIV-1 infection requires CPSF6
Source: Retrovirology. 2013 Apr 26;10:46. doi: 10.1186/1742-4690-10-46 (PMC3695788; doi:10.1186/1742-4690-10-46)
Supplement: Additional file 2 — Subcellular localization of CPSF6 transfected in the different cell lines. [file 1742-4690-10-46-S2.pdf]

	Subcellular localization of CPSF6 transfected in the different cell lines								
	Experiment 1			Experiment 2			Experiment 3		
HeLa cells	Exclusively nuclear	Exclusively cytoplasm	Throughout cell	Exclusively nuclear	Exclusively cytoplasm	Throughout cell	Exclusively nuclear	Exclusively cytoplasm	Throughout cell
shRNA control	193	0	7	185	0	15	190	0	10
TNPO3 K.D.	181	0	19	177	0	23	175	0	25

	Subcellular localization of ASF/SF2 transfected in the different cell lines								
	Experiment 1			Experiment 2			Experiment 3		
HeLa cells	Exclusively nuclear	Exclusively cytoplasm	Throughout cell	Exclusively nuclear	Exclusively cytoplasm	Throughout cell	Exclusively nuclear	Exclusively cytoplasm	Throughout cell
shRNA control	193	0	7	173	0	27	189	0	11
TNPO3 K.D.	21	0	179	24	0	176	33	0	167

AF2
