# Supplementary material for: The ability of TNPO3-depleted cells to inhibit HIV-1 infection requires CPSF6
Source: Retrovirology. 2013 Apr 26;10:46. doi: 10.1186/1742-4690-10-46 (PMC3695788; doi:10.1186/1742-4690-10-46)
Supplement: Additional file 3 — Subcellular localization of CPSF6. [file 1742-4690-10-46-S3.pdf]

	Subcellular localization of CPSF6								
	Experiment 1			Experiment 2			Experiment 3		
**Cf2Th cells**	**Exclusively nuclear**	**Exclusively cytoplasm**	**Throughout cell**	**Exclusively nuclear**	**Exclusively cytoplasm**	**Throughout cell**	**Exclusively nuclear**	**Exclusively cytoplasm**	**Throughout cell**
CPSF6	197	0	3	193	0	7	189	0	11
CPSF6-FG284AA	195	0	5	198	0	2	192	0	8
NES-CPSF6	0	110	90	0	112	88	0	111	89
NES-CPSF6-FG284AA	0	105	95	0	103	97	0	114	86

AF3
