# Supplementary material for: The ability of TNPO3-depleted cells to inhibit HIV-1 infection requires CPSF6
Source: Retrovirology. 2013 Apr 26;10:46. doi: 10.1186/1742-4690-10-46 (PMC3695788; doi:10.1186/1742-4690-10-46)
Supplement: Additional file 4 — Subcellular localization of CPSF6. [file 1742-4690-10-46-S4.pdf]

	Subcellular localization of CPSF6								
	Experiment 1			Experiment 2			Experiment 3		
**Hela cells**	**Exclusively nuclear**	**Exclusively cytoplasm**	**Throughout cell**	**Exclusively nuclear**	**Exclusively cytoplasm**	**Throughout cell**	**Exclusively nuclear**	**Exclusively cytoplasm**	**Throughout cell**
CPSF6	195	0	5	192	0	8	187	0	13
CPSF6-FG284AA	190	0	10	193	0	7	191	0	9
NES-CPSF6	0	112	88	0	120	80	0	113	87
NES-CPSF6-FG284AA	0	107	93	0	102	98	0	115	85

AF4
